# Supplementary material for: Protein interface redesign facilitates the transformation of nanocage building blocks to 1D and 2D nanomaterials
Source: Nat Commun. 2021 Aug 11;12:4849. doi: 10.1038/s41467-021-25199-x (PMC8357837; doi:10.1038/s41467-021-25199-x)
Supplement: Supplementary file 1 — Supplementary Information [file 41467_2021_25199_MOESM1_ESM.pdf]

Supplementary Information

Protein interface redesign facilitates the transformation of nanocage building blocks to 1D and 2D nanomaterials

Xiaorong Zhang[1], Yu Liu[1], Bowen Zheng[1], Jiachen Zang[1], Chenyan Lv[1], Tuo Zhang[1,*], Hongfei Wang[2], Guanghua Zhao[1,*]

[1]*College of Food Science & Nutritional Engineering, China Agricultural University, Beijing Key Laboratory of Functional Food from Plant Resources, Beijing 100083, China*

[2]*Key Laboratory of Chemical Biology and Molecular Engineering of Education, Ministry, Key Laboratory of Energy Conversion and Storage Materials of Shanxi Province, Institute of Molecular Science, Shanxi University, Taiyuan 030006, China*

*Corresponding author: Tuo Zhang, E-mail: zhangtuo@cau.edu.cn; Guanghua Zhao, E-mail: gzhao@cau.edu.cn, College of Food Science and Nutritional Engineering, China Agricultural University, Beijing 100083, China.

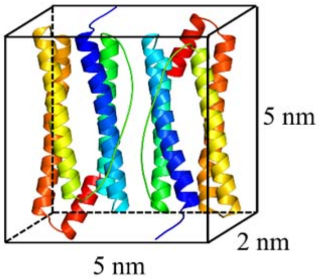

Supplementary Figure 1. The crystal structure of TmFtn. The dimeric TmFtn can be simulated as a cuboid with 5 nm in length, 2 nm in width and 5 nm in height.

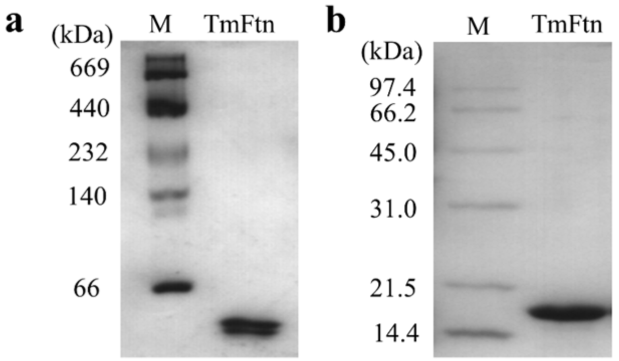

Supplementary Figure 2. Preparation of TmFtn. Native-PAGE (a) and SDS-PAGE (b) analyses of purified TmFtn. Lane M, protein markers and their corresponding molecular masses. The molecular weight of TmFtn subunit is approximately 20 kDa.

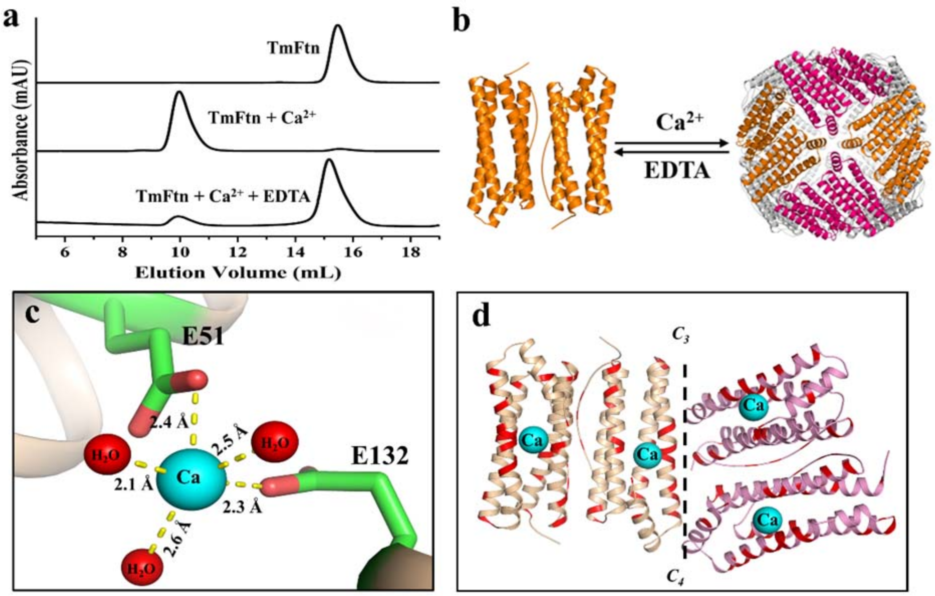

Supplementary Figure 3. Characterization of the reversible self-assembly property of *Thermotoga maritima* ferritin (TmFtn) induced by calcium ions. (a) High-resolution gel filtration chromatography analyses revealed that dimeric TmFtn assemble into 24-meric protein nanocage in the presence of Ca^{2+}, while the formed protein nanocage disassembles into dimers upon treatment with EDTA. Conditions: [TmFtn] = 12.0 μM buffered in 50 mM Tris-HCl, pH = 8.0; [Ca^{2+}] = 50 mM; [EDTA] = 100 mM. (b) Schematic showing the reversible assembly of TmFtn. (c) Close-up view of Ca^{2+} coordination with Glu51, Glu132 and three H_2O molecules. (d) The coordinated Ca^{2+} ions nearby the C_3-C_4 interface counteract the negative charges, thereby promoting the formation of 24-meric protein nanocage. Residues with negative charges were highlighted in red.

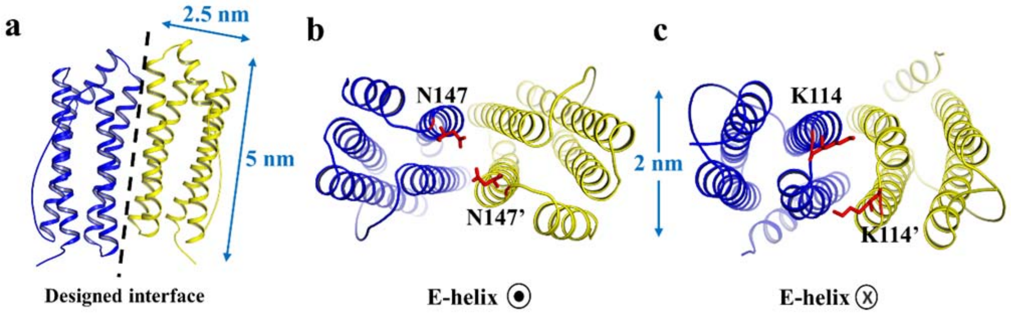

Supplementary Figure 4. The design of protein interface. (a) The Rosetta-calculated model of the interaction of two adjacent dimeric proteins in a fully side-by-side manner. (b, c) Predicted surface residues suitable for the introduction of noncovalent interactions. Dimers are represented in different colors, and each dimer only shows one subunit.

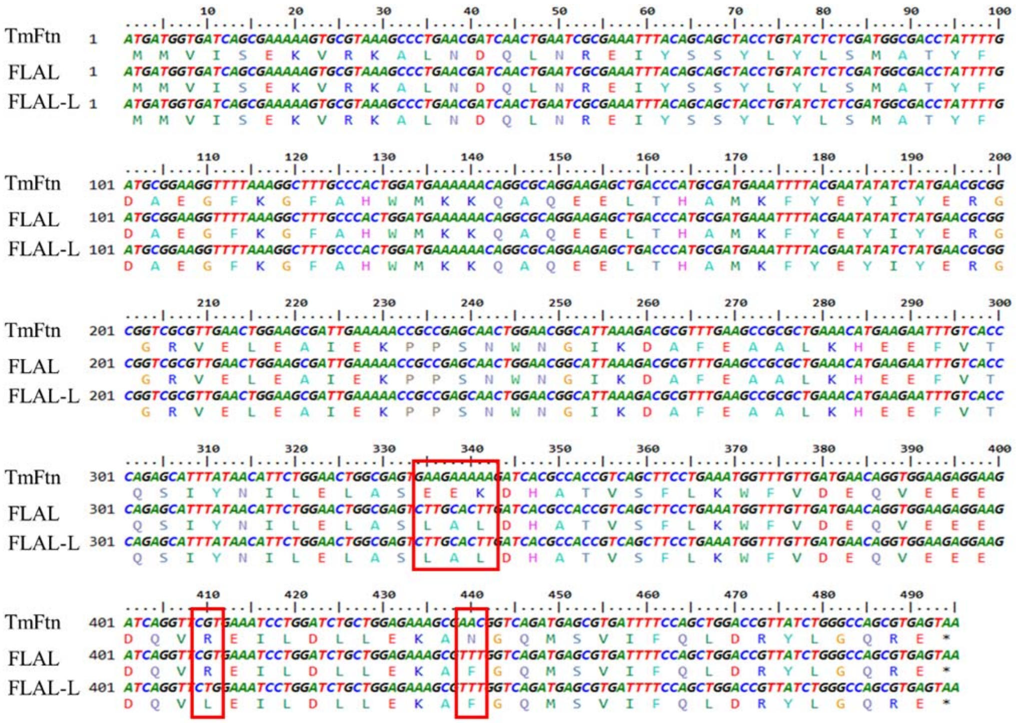

Supplementary Figure 5. Sequence alignment of TmFtn, FLAL and FLAL-L.

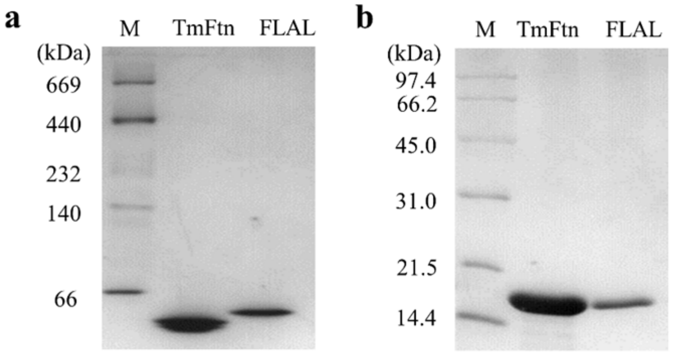

Supplementary Figure 6. Preparation and characterization of FLAL. Native PAGE (a) and SDS-PAGE (b) analyses of purified TmFtn and FLAL. Lane M, protein markers and their corresponding molecular masses.

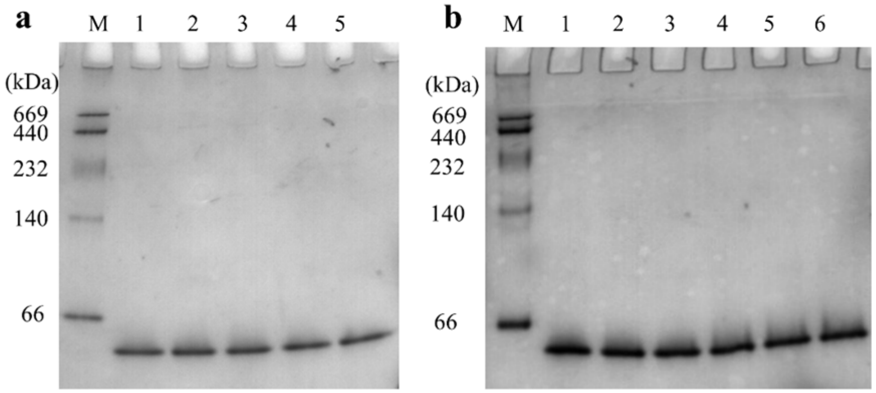

Supplementary Figure 7. The charactization of FLAL in solution under different conditions. (a) The components of FLAL solution at different pH values were analyzed by native PAGE. Lane 1, pH 6.0; lane 2, pH 7.0; lane 3, pH 8.0; lane 4, pH 9.0; lane 5, pH 10.0; and lane M, protein markers and their corresponding molecular masses. Conditions: [FLAL] = 12.0 μM. (b) The components of FLAL solution with different concentrations of NaCl were analyzed by native PAGE. Lane 1, 0 mM NaCl; lane 2, 100 mM NaCl; lane 3, 200 mM NaCl; lane 4, 300 mM NaCl; lane 5, 400 mM NaCl; lane 6, 500 mM NaCl; and lane M, protein markers and their corresponding molecular masses. Conditions: [FLAL] = 12.0 μM buffered in 50 mM Tris-HCl, pH = 8.0.

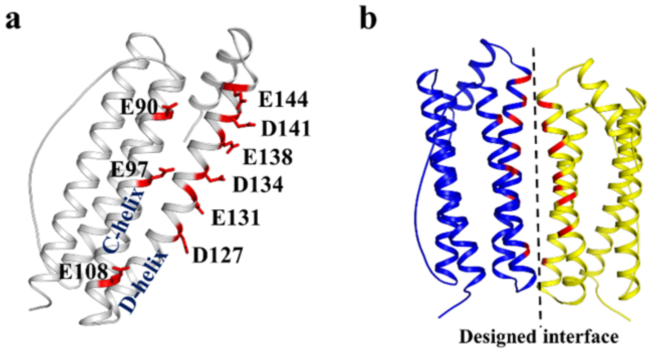

Supplementary Figure 8. Amino acid residues analyses along the designed interface. C-helix and D-helix of Tmftn contains many acidic amino acid residues (a), which generate electrostatic repulsion on the designed protein-protein interface (b), and thus impede FLAL molecules assembly into 1D nanostructure. Residues with negative charges were highlighted in red.

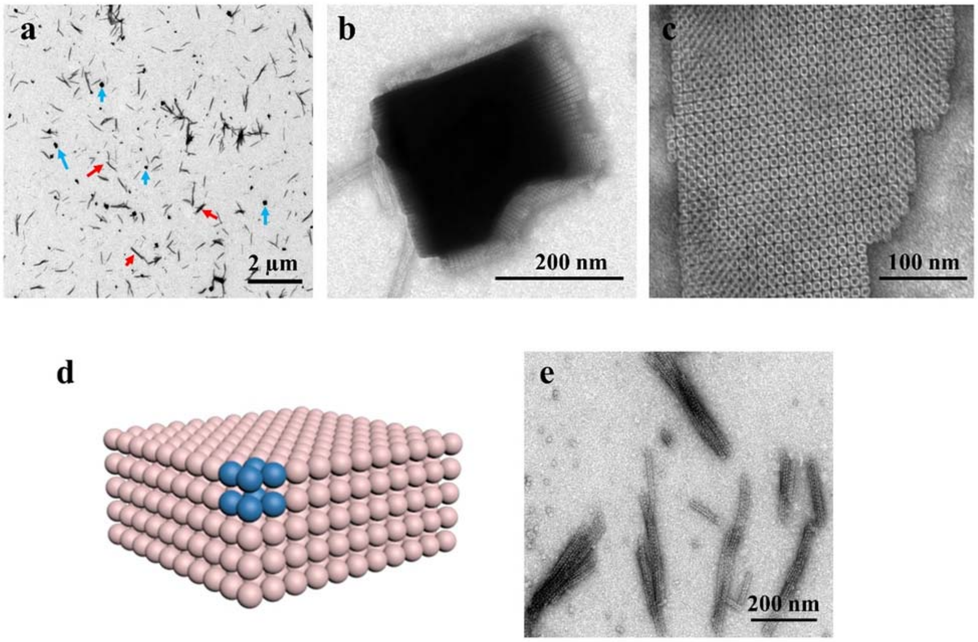

Supplementary Figure 9. Self-assembly of FLAL induced by calcium ions. (a) Low-magnification TEM images of self-assembled products of the mutant FLAL upon treatment with Ca^{2+} for 30 min. (b, c) Enlargements of the product corresponding to the product marked by the blue arrows in (a). (d) Structural model of the simple cubic architecture corresponding to panel b. (e) High-magnification TEM view of the product corresponding to the product marked by the red arrows in (a). Conditions: [FLAL] = 48 μM in 50 mM Tris-HCl, pH 8.0, [Ca^{2+}] = 80 mM.

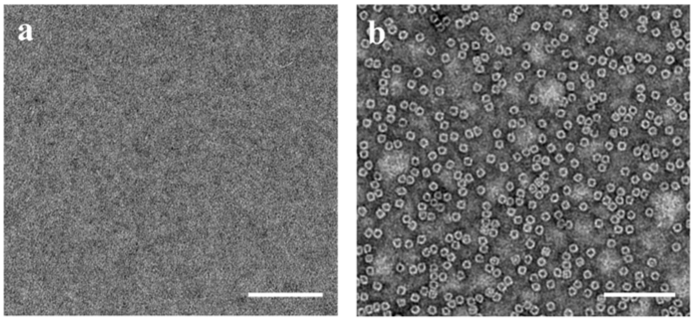

Supplementary Figure 10. Self-assembly analyses of natural TmFtn. TEM images of TmFtn in the absence (a) and presence of 80 mM Ca^{2+} (b). Conditions: [TmFtn] = 48.0 μM in 50 mM Tris-HCl, pH 8.0, [Ca^{2+}] = 80 mM. (a, b) Scale bars represent 100 nm.

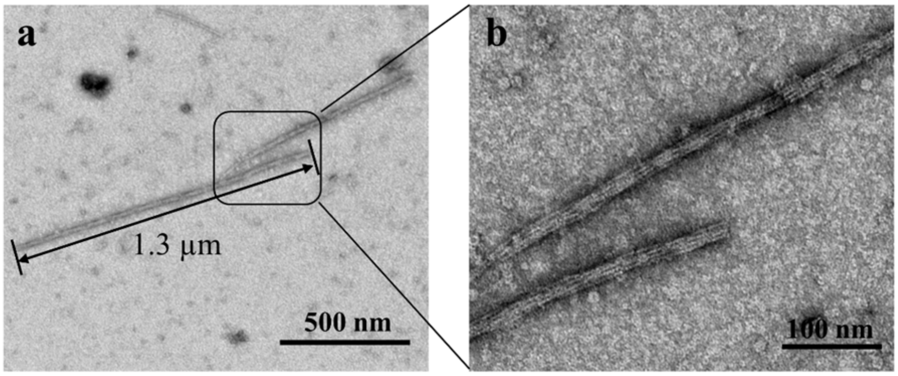

Supplementary Figure 11. The characterization of FLAL fliaments. (a) TEM images of fliaments constructed by 48.0 μM of FLAL and 80 mM of Ca^{2+}, and the length of the filamnet can reach 1.3 μm. (b) Enlargement of the filament shown in panel a.

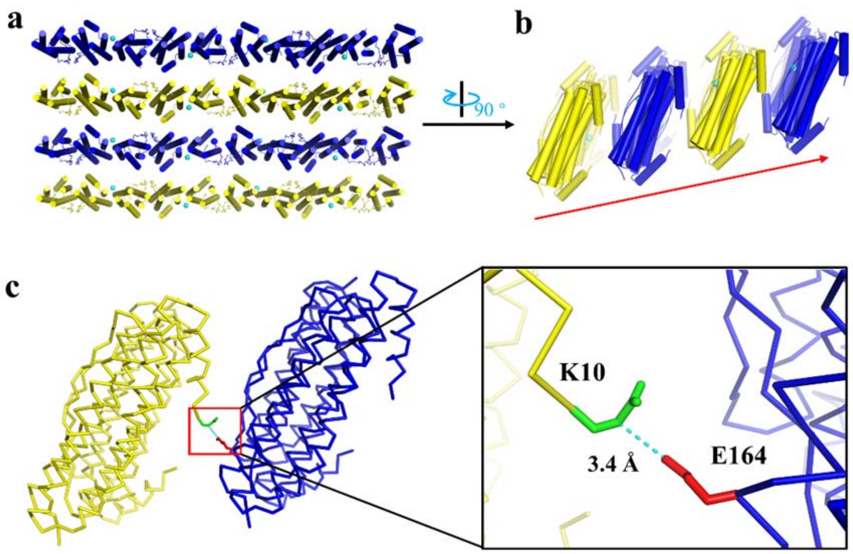

Supplementary Figure 12. The arrangement of FLAL filaments. (a) In the crystal structure, the formed 1D filaments further arrange in the vertical direction to create 2D protein assemblies. (b) From the side view, 1D Filaments are parallel-displaced, red arrow represents the direction of the filament. (c) Two adjacent filaments are connected by electrostatic attraction between K10 and E164.

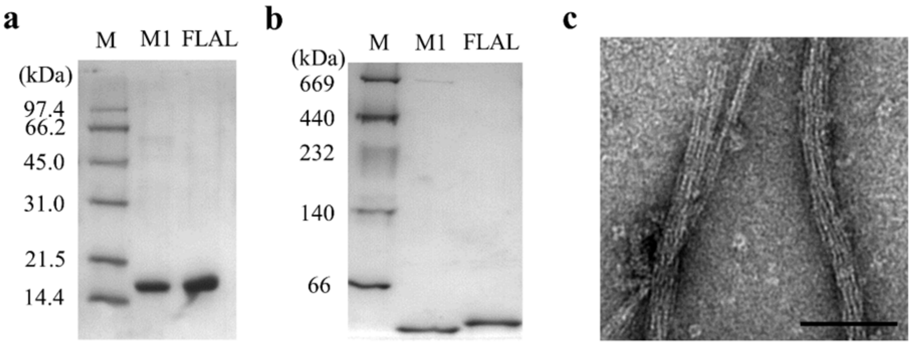

Supplementary Fig. 13. Preparation and characterization of mutant M1. SDS-PAGE (a) and Native-PAGE (b) analyses of mutant M1. Lane M, protein markers and their corresponding molecular masses. (c) TEM images of fliaments constructed by 48.0 μM of the muatnt M1 and 80 mM Ca^{2+}. The scale bar represents 100 nm.

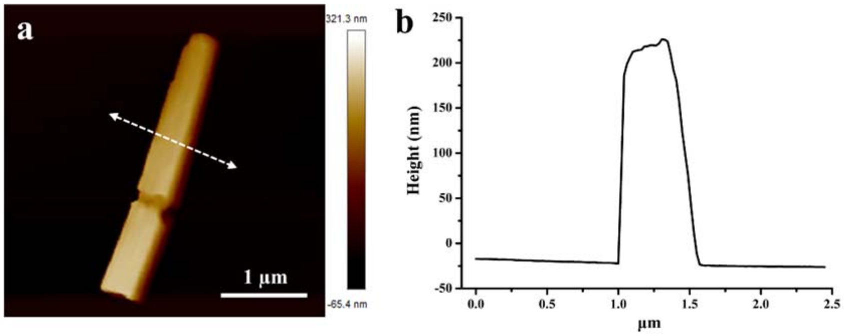

Supplementary Figure 14. Atomic force microscopy (AFM) micrographs of FLAL nanorod. (a) Large view of the nanorod. (b) Height distributions measured along the white two-way arrows in (a).

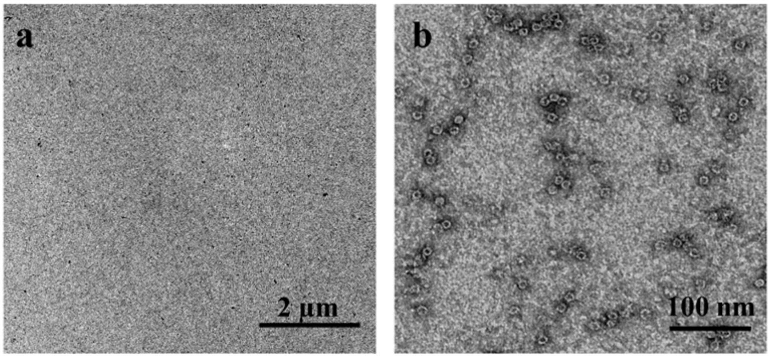

Supplementary Figure 15. Effect of PEG1500 on TmFtn self-assemby. Low (a) and high (b) magnification TEM view of TmFtn in the presence of 30% PEG1500. Conditions: [TmFtn] = 12.0 μM in 50 mM Tris-HCl, pH 8.0.

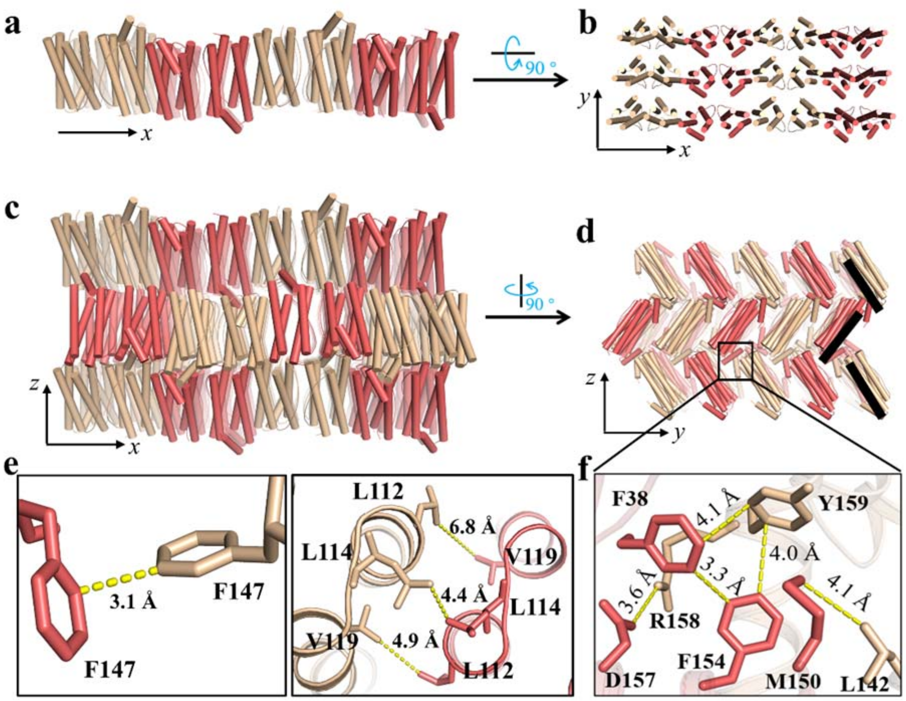

Supplementary Figure 16. The crystal structure of FLAL in the presence of PEG1500. (a) In the crystal structure, FLAL molecules are positioned in a fully side-by-side manner along the x-axis to form 1D arrays. (b) These 1D arrays are stacked along the y-axis to form 2D arrays. (c) The formed 2D arrays further arrange in the vertical direction to create 3D protein assemblies. (d) Another perspective of (c). (e) Close-up views of the designed interfacial interactions in the 1D arrays. (f) A Close-up view of the noncovalent interactions that drive the stacking of the 2D arrays.

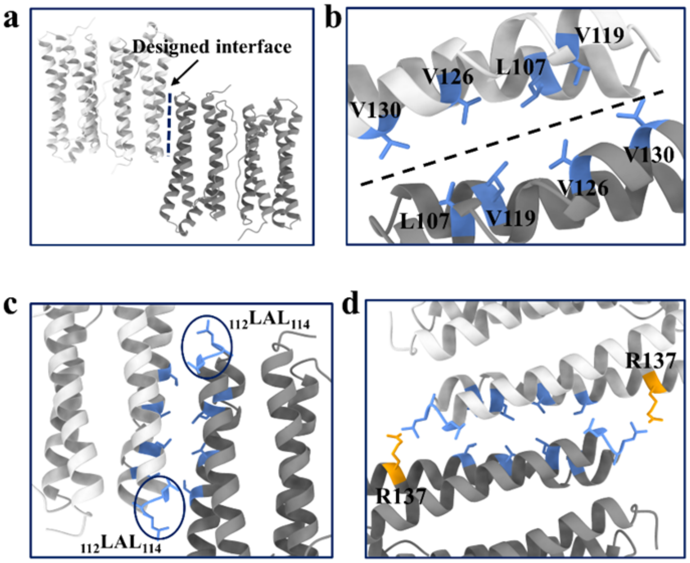

Supplementary Figure 17. Analyses of TmFtn dimer arranged in a partially side-by-side model. (a) The Rosetta-calculated model of the interaction of two contiguous dimeric TmFtn molecules in a partially side-to-side manner. (b) The hydrophobic amino acid residues at the designed interface. (c) The Rosetta-calculated model of the interaction of two contiguous FLAL dimers in a partially side-to-side manner. Because of the existence of three more hydrophobic residues in FLAL than TmFtn, the hydrophobic interactions at the interface between two adjacent dimeric FLAL molecules is stronger than those between two adjacent dimeric TmFtn molecules. (d) Subsititution of Arg137 with leucine in FLAL can increase the area of the hydrophobic core formed along the designed interface.

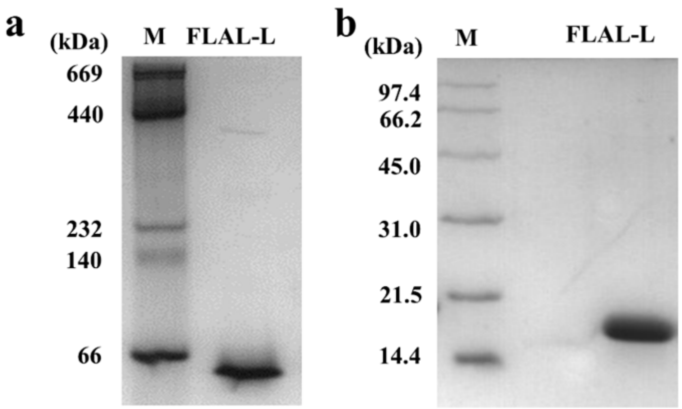

Supplementary Figure 18. Preparation and characterization of mutant FLAL-L. Native PAGE (a) and SDS-PAGE (b) analyses of purified mutant FLAL-L. Lane M, protein markers and their corresponding molecular masses.

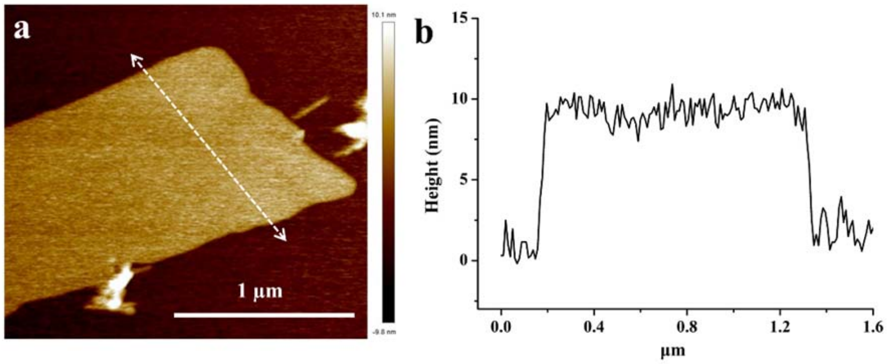

Supplementary Figure 19. Atomic force microscopy (AFM) micrographs of 2D FLAL-L nanoribbon. (a) Large view of the nanoribbon. (b) Height distributions measured along the white two-way arrows in (a).

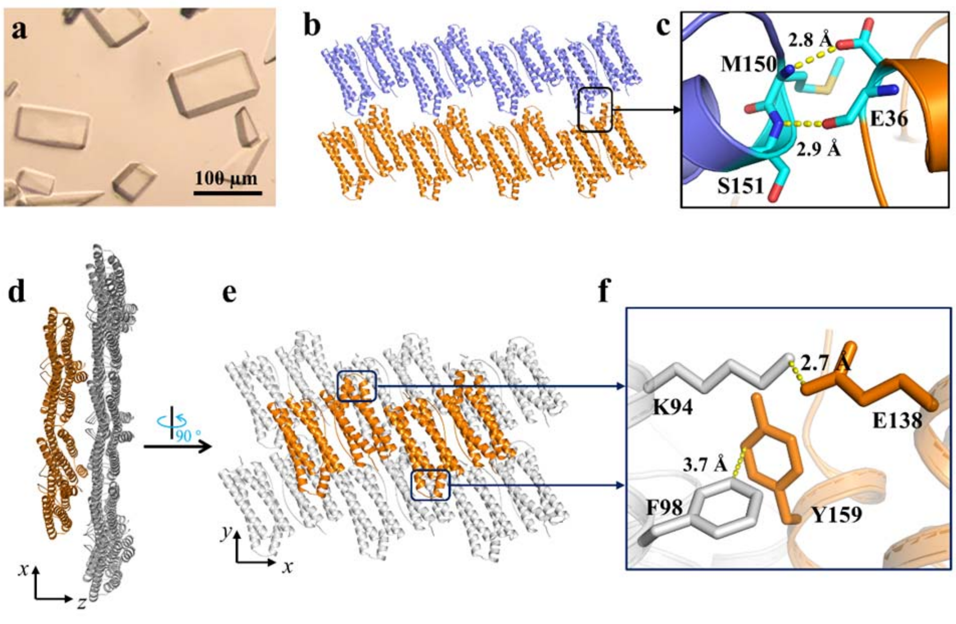

Supplementary Figure 20. Detailed noncovalent interactions in FLAL-L nanoribbons. (a) Optical microscope image of crystals of FLAL-L. (b-c) In the crystal structure of FLAL-L, 1D filaments coalesce in parallel along the *y* axes through hydrogen bonds to form 2D protein layers. (d-f) The formed 2D protein layers further arrange along the *z* axes to form 3D protein frameworks through π-π interactions and electrostatic interactions.

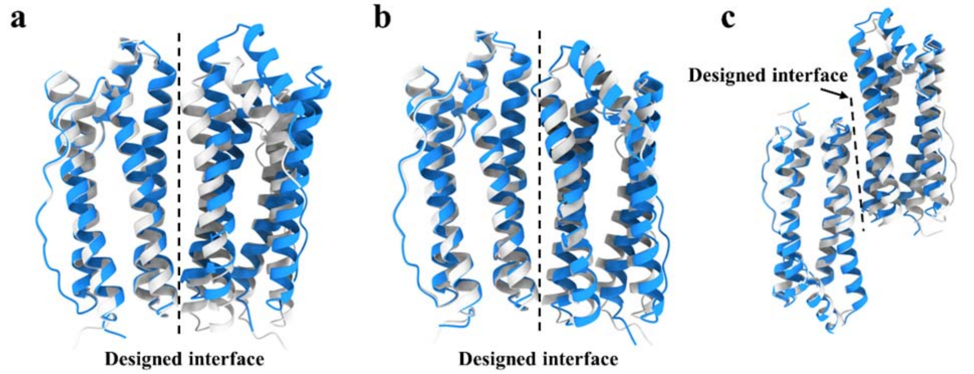

Supplementary Figure 21. Comparison of the computational design models (gray) and the X-ray crystal structures (dodger blue). (a) Ca^{2+}-induced FLAL assembly. (b) PEG-induced FLAL assembly. (c) PEG-induced FLAL-L assembly.

	Crystallization conditions	pH
TmFtn-cage	14% 2-Propanol, 70 mM Sodium acetate/ HCl, 140 mM Calcium chloride, 30% Glycerol	4.6
FLAL①	0.1M $Ca(Ac)_2$, 20% PEG 1000, and 100 mM Imidazole/HCl	7.0
FLAL②	30% PEG 1500	8.0
FLAL-L	200 mM NaCl, 20% PEG 3000 and 100 mM HEPES/NaOH	7.5

Supplementary Table 1 Crystallization conditions for each crystal.

Supplementary Table 2 Crystallographic statistics.

Parameters	TmFtn-cage	FLAL①	FLAL②	FLAL-L
**Data collection**				
Wavelength (Å)	0.980	0.979	0.979	0.989
Space group	H32	$P2_12_12_1$	$P4_22_12$	C2
Unit cell				
a, b, c (Å)	175.434 175.434 357.421	39.418 85.981 107.09	224.39 224.39 131.76	175.343 54.647 59.91
α , β, γ (°)	90 90 120	90 90 90	90 90 90	90, 109.943, 90
[a]Resolution (Å)	38.51-2.197 (2.276-2.197)	29.05-2.103 (2.179-2.103)	22.45-2.64 (2.734-2.64)	56.32-1.78 (1.844-1.78)
[a]Completeness (%)	99.92 (100)	98.44 (100)	95.24 (100)	94.64 (100)
Mean I/sigma(I)	2.75 (2.20)	5.63 (2.10)	2.83 (2.32)	2.50 (1.78)
[b]$CC_{1/2}$ of the highest resolution shell	0.992 (979)	0.982 (0.969)	0.997 (0.935)	0.997 (0.947)
**Refinement**				
Unique reflections	107433 (10652)	21568(2073)	95474(9361)	48748(3604)
Reflections used in refinement	20904(2061)	21539(2073)	93941(9316)	48600(3596)
Reflections used for R-free	1999(198)	1997(192)	4699(479)	2008(150)
Non-hydrogen Atoms	11989	2773	16332	4536
Protein residues	1312	325	1967	489
Wilson B-factor ($Å^2$)	35.15	32.08	32.08	21.48
[c]R_{work} (%)	0.1747 (0.1987)	0.2101(0.2423)	0.3386(0.3435)	0.1945(0.2913)
[d]R_{free} (%)	0.2007 (0.2267)	0.2637(0.3331)	0.4048(0.3903)	0.2346(0.3590)
macromolecules	10945	2723	2723	4075
ligands	25	10	12	6
RMS(bonds)	0.008	0.008	0.009	0.11

RMS(angles)	1.01	0.81	1.18	1.57
Ramachandran favored (%)	99.07	98.44	97.68	98.76
Ramachandran allowed (%)	0.93	1.56	2.01	1.04
Ramachandran outliers (%)	0.00	0.00	0.31	0.21

[a]Highest resolution shell is shown in parentheses.

[b]CC1/2 is the correlation coefficient of the half datasets.

[c]$R_{work} = \sum_{hkl} \| |F_{obs}| - |F_{calc}| \| / \sum_{hkl} |F_{obs}|$, where Fobs and Fcalc is the observed and the calculated structure factor, respectively.

[d]Rfree is the cross-validation R factor for the test set of reflections (5% of the total) omitted in model refinement.

Supplementary Table 3 Behaviors of FLAL mutant at different concentrations of FLAL and Ca^{2+}.

		Ca^{2+} concentration				
		20 mM	40 mM	60 mM	80 mM	100 mM
Protein concentration	12 µM	N	N	N	N	A
	24 µM	N	N	N	N	A
	36 µM	A	A	A	F	F
	48 µM	A	A	F	F	F
	60 µM	A	A	F	F	F

N - No assembly; A – Aggregation; F - Filament.

Supplementary Table 4 Different behaviors of FLAL-L mutant at different PEG concentrations and molecular weights.

	PEG concentration			
	5%	10%	15%	20%
PEG 400	N	N	N	N
PEG 1000	A	A	A	A
PEG 1500	A	A	F	F
PEG 3000	A	A	A	A

N - No filament; A - Aggregation; F - Filament.

Supplementary Table 5 Primers used in mutants preparation.

names	mutation site	primers
FLAL	N147F	5’-GATCTGCTGGAGAAAGCGTTTGGTCAGATGAGCGTGATT-3’<br>3’-CTAGACGACCTCTTTCGCAAACCAGTCTACTCGCACTAA-5’
	$_{112}$EEK$_{114}$—$_{112}$LAL$_{114}$	5’-ATTCTGGAACTGGCGAGTCTTGCACTTGATCACGCCACCGTCAGC-3’<br>3’-TAAGACCTTGACCGCTCAGAACGTGAACTAGTGCGGTGGCAGTCG-5’
FLAL-L	R137L	5’-GAAGAGGAAGATCAGGTTCTGGAAATCCTGGATCTGCTG-3’<br>3’-CTTCTCCTTCTAGTCCAAGACCTTTAGGACCTAGACGAC-5’
M1	K10G	5’-ATCAGCGAAAAAGTGCGTGGCGCCCTGAACGATCAACTG-3’<br>3’-TAGTCGCTTTTTCACGCACCGCGGGACTTGCTAGTTGAC-5’
	E164G	5’-CGTTATCTGGGCCAGCGTGGCTAAGGATCCGGCTGCTAA-3’<br>3’-GCAATAGACCCGGTCGCACCGATTCCTAGGCCGACGATT-5’
